# Dissemination of circulating tumor cells at night: role of sleep or circadian rhythm?

**DOI:** 10.1186/s13059-022-02791-y

**Published:** 2022-10-14

**Authors:** Yves Dauvilliers, Frédéric Thomas, Catherine Alix-Panabières

**Affiliations:** 1 Department of Neurology, Sleep Troubles and Narcolepsy, University Hospital of Montpellier, Montpellier, France; 2 CREEC/CANECEV, MIVEGEC (CREES), University of Montpellier, CNRS, IRD, Montpellier, France; 3 Laboratory of Rare Human Circulating Cells (LCCRH), University Hospital of Montpellier, 641, avenue du Doyen Gaston Giraud, 34093 Montpellier Cedex 5, France

**Keywords:** Circulating tumor cells, Sleep, Circadian cycle, Metastasis

The analysis of blood for circulating tumor cells (CTCs) called *liquid biopsy* has opened new avenues for cancer diagnostic and management, as well as the comprehension of the metastatic cascade [1]. As metastasis is responsible for about 90% of cancer deaths [2], it is of utmost importance to decrease the survival of CTCs, mostly to target specifically the metastasis-competent CTCs while they are reaching distant organs [3].

*How CTCs are disseminating through the bloodstream?* In advanced cancers, CTCs can travel as clusters or microemboli in the blood. CTCs traveling in a group seem to be released from hypoxic tumor regions [4] and clearly present a specific hypomethylation pattern related to stem cell features, underlying their higher aggressiveness [5]. Consequently, CTC clusters predict a poorer prognosis compared to single CTCs. Interestingly, CTC microemboli can also include other cell types, such as immune cells and/or cancer-associated fibroblasts from the tumor microenvironment, which have a higher capacity to survive and to metastasize.

*When CTCs are traveling the most?* This specific question has already arised in 2020 when Cortés-Hernández et al. hypothesized that, because of evolutionary ecology reasons, malignant cells, including CTCs, should behave differently between day and night [6]. The recent work of Diamantopoulou et al. showed that the levels of CTCs increased at night in humans with breast cancer and mouse models of breast cancer and that CTCs in the rest-phase are more likely to metastasize [7]. This non-continuous generation of CTCs within the rest phase, if confirmed, will undoubtedly have major medical implications. However, key questions remain at this stage: Is it sleep and/or the circadian rhythm involved in the formation of CTCs and the metastatic spread of cancer, and by which specific mechanisms?

The biology of most, if not all, living things is regulated by biological rhythms, in particular sleep and the circadian rhythm [8]. Due to the extreme diversity of cells typically generated in tumors, natural selection acting on cells, and/or groups of cells, may also promote the emergence of clones that exhibit, for each stage of tumorigenesis, the best characteristics for a malignant lifestyle, thus allowing the tumor to continue its progression to the metastatic stage. Several tumors are capable of releasing CTCs, but those with the characteristics found by Diamantopoulou et al., during the rest phase, efficiently produce metastasis. There are likely many reasons why malignant cells can be influenced by sleep and the circadian rhythm [6]; however, the findings that CTCs were reported at highest levels at night and were more prone to metastasize remain to be better understood. Circadian rhythms and sleep are fundamental biological processes that regulate physiology and behavior, including hormone secretion, metabolism, DNA repair, and apoptosis [9]. Disruption of circadian rhythms and sleep promote several disturbances, ranging from abnormal vigilance, altered metabolism, heart disease, reduced immunity, increased stress and mood states, and cancer [9]. Precisely, a pooled analysis of population-based case-control studies reported that premenopausal women night workers were associated with an increased risk of breast cancer, particularly when working for 3 or more nights per week for at least 10 years [10].

**Fig. 1:**
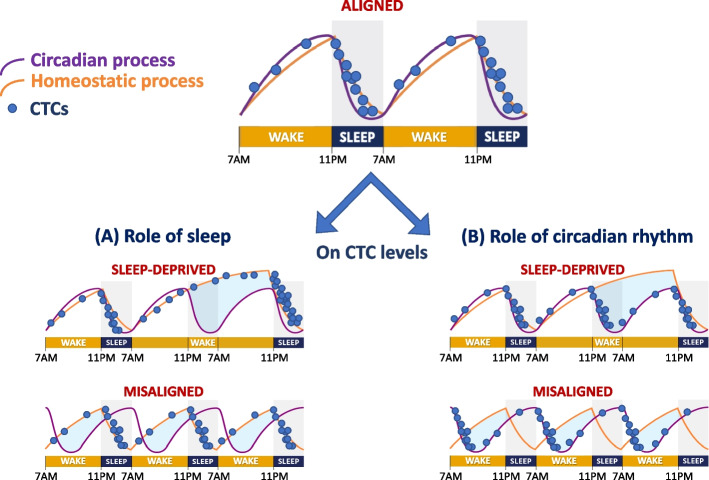
The two-process model of sleep is mediated by the interaction between a homeostatic process (*curve in green*) that determines the rise of sleep pressure during the waketime and its fall during sleep and a circadian process (*curve in red*) that follows a 24-h rhythm driven by the suprachiasmatic nucleus, the master pacemaker. The temporal evolution of homeostatic and circadian processes is most often aligned but can be disturbed particularly in sleep deprived conditions and misalignment (e.g., jet lag, shift work, delayed/advanced clock). As a result, sleep will be misaligned with the circadian rhythm (e.g., rise vs fall in body temperature). It is unclear whether circulating tumor cells (CTCs, *blue circles*) increased due to sleep or circadian phase. Two hypotheses have been illustrated: **A** the impact of sleep with elevated CTC levels during sleep increasing in sleep-deprived condition and with separation from the circadian rhythm in the misaligned condition and **B** the impact of the circadian rhythm with high levels of CTCs during the circadian phase (i.e., fall in body temperature) that do not follow the sleep pattern during sleep deprivation and misaligned conditions. This figure has been modified from Lane et al. [12]. CTCs, circulating tumor cells

Assessing by two blood samples the percentage of single CTCs, homotypic CTC clusters and heterotypic CTC-white blood cells in rest (at 4:00 am) vs active (at 10:00 am) phase in 30 women with breast cancer at different stages of the disease, the recent study found that 78.3% of CTCs were obtained at nighttime [7]. While the 4:00 am often corresponds to the nadir of the circadian rhythm, 10:00 am does not correspond to its peak. It would have been accurate to take the samples 12 h apart and measured them in linked to endogenous circadian biomarkers (e.g., core body temperature, hormonal secretion) and to the chronotype of patients (evening vs morning type). Moreover, sleep and circadian rhythm are different phenomena, often acting in concert but sometimes with dissociated patterns which can have consequences on the spontaneous CTCs generation (Fig. 1). The role of sleep per se was not investigated in this study although reported in the title of the manuscript. Cancer cells spread aggressively during the resting phase; however, whether this process occurs during sleep phases, non-rapid eye movement (NREM), or rapid eye movement (REM) sleep remains to be explored. Several studies suggest that patients with breast cancer had comorbid insomnia, reduced sleep times, frequent sleep disturbances, and sometimes occurred in the context of prolonged nightshift work. A recent study showed that night work was associated with a decrease in the circadian rhythm in half of health professionals which may persist in days off in some of them [11]. The rhythms of sleep-wake (e.g., regular sleep regime, bedtime and wake up time routine), sleep duration, and the presence of sleep disorders including insomnia and related central nervous system drugs intake should be assessed in women with breast cancer.

Mouse models of breast cancer have also shown increased levels of CTCs in the resting phase of the circadian rhythm, and a shift in the normal light/dark cycle causing a jet-lag effect markedly decreased CTC level. In addition, treatment with melatonin 2 h before the start of the rest phase to regulate the sleep cycle clearly induced the production of CTCs. However, no sleep deprivation experiments, no sleep rebound studies, no distinction between NREM and REM sleeps, and any studies of the effects of wake-promoting or hypnotic agents have been performed to date. Finally, Diamantopoulou et al. also reported that CTCs from the rest phase form tumors that are more aggressive than those from the active phase in humans and mice, findings that may be linked to change in gene expression associated with cell proliferation. Increased expression of glucocorticoid, androgen, and insulin receptors was also noted in CTCs, with prolonged CTCs suppression after treatment with dexamethasone, testosterone, and insulin.

It is timely to determine whether sleep per se or circadian rhythm influences the dynamics of CTCs by performing repeated night and day sampling coupled with sleep-wake recording in the future and whether this is confirmed in other hormono-dependent cancers or not. Sleep disturbances are common in people affected by cancer and for reasons that are so far unclear. Alteration in the circadian rhythm or in the sleep-wake pattern may be additional warning signals of poor health associated with cancer. This is a prerequisite before considering a precise chronotherapy (e.g., schedule for taking CTCs samples and taking chemotherapy) or even evaluating the impact of drugs that can regulate sleep or circadian rhythms in the genesis of CTCs and cancer progression.

In conclusion, we need to put more effort on dissecting the metastatic cascade including the dynamics of the CTCs depending on the sleep and/or the circadian cycle in patients with solid cancers. We also need to better understand how sleep and circadian rhythm differentially influence the host defense efficiency against cancer to better predict which of these two processes is the most likely to shape cancer cell activities. The future will tell us whether there is a specific timing when to administrate the treatment to patients. *In fine*, clinical practice should use precision medicine for cancer patients as “the right drug, to the specific patient, on the right day and the best timing during this day or night.”
